# Surgical Drain-Related Small Bowel Obstruction After Open Radical Cystoprostatectomy: A Case Report

**DOI:** 10.7759/cureus.46368

**Published:** 2023-10-02

**Authors:** Xin Lin, Jennifer Lu, John Fitzgerald

**Affiliations:** 1 Urology, New York Institute of Technology College of Osteopathic Medicine, Old Westbury, USA; 2 Urology, School of Medicine, Stony Brook University, Stony Brook, USA

**Keywords:** bladder cancer, postoperative ileus, radical cystectomy, small bowel obstruction, surgical drains

## Abstract

Surgical drains are commonly used to manage intraperitoneal fluid after major surgeries, but their prophylactic use has been controversial due to potential complications. One rarely reported complication is small bowel obstruction (SBO), primarily seen in post-colorectal surgeries. We present a case of SBO following open radical cystectomy due to surgical drain placement, a complication not previously reported in urologic surgeries. The case highlights the importance of considering the risks and benefits of prophylactic drain placement. It emphasizes the need for a higher index of suspicion for SBO in patients with surgical drains who develop post-operative nausea and distention. Timely radiological imaging and clinical examination are crucial for accurate diagnosis and proper treatment.

## Introduction

Surgical drains are commonly placed within the abdominal and pelvic cavity after major surgeries, including cystectomy, for drainage of excess intraperitoneal fluid collection. However, the use of prophylactic drainage has been a controversial topic due to potential complications such as leaks and infections [1]. A rarely reported complication of surgical drain placement is the development of small bowel obstruction (SBO), which is mostly noted in post-colorectal surgeries. SBO is a common gastrointestinal complication often caused by mechanical obstructions such as intraabdominal adhesions. Symptoms often include nausea, vomiting, abdominal distension, and obstipation due to the partial or complete blockage of the small intestine [2]. Symptoms of SBO occurring within 30 days of initial surgery are considered early SBO. Beyond that time frame, they are categorized as late SBO. In the field of urology, early SBO is a potential complication after any major open or laparoscopic surgery, with the most common cause being the development of adhesions [3]. However, SBO caused by surgical drain following radical cystectomy has never been reported.

## Case presentation

A 79-year-old male with an extensive past medical history, including prostate cancer treated with radiation in 2008 and carcinoma in situ of the bladder first diagnosed in 2016, underwent treatment with BCG, followed by mitomycin, before being lost to follow-up. In November 2022, he underwent a blue light cystoscopy, which revealed a large lobulated lesion on the left trigone of the bladder. The patient had undergone transurethral resection of a large lesion and left ureteral stent insertion of left hydroureteronephrosis seen on retrograde pyelogram. The pathology resulting from this procedure revealed high-grade urothelial carcinoma invading the muscularis propria. The patient was duly informed of the risks, benefits, and alternative treatment options available for muscle-invasive bladder cancer. After considering these, the patient chose to proceed with a radical cystoprostatectomy with urinary diversion. The patient underwent an open radical cystoprostatectomy, bilateral pelvic lymph node dissection, and the creation of an ileal conduit with ileal-ureteral anastomosis on January 16, 2023. The final pathology report revealed an ulcerated, high-grade urothelial carcinoma that invaded through the muscularis propria of the bladder, with surgical resection margins being negative. Additionally, bilateral lymph nodes were examined and were found to be negative for metastatic carcinoma. As part of the standard post-operative care, bilateral ureteral stents were inserted through the ureteral anastomosis, and a Foley catheter was inserted per urethra for pelvic drainage. A 19 French Blake surgical drain was introduced into the pelvic cavity through a stab incision in the left lower quadrant via the lateral abdominal wall, and a nasogastric tube (NGT) was placed. The positioning of all tubes was confirmed, with a post-operative abdominal X-ray demonstrating proper placement. All drainage output was monitored daily.

**Figure 1:**
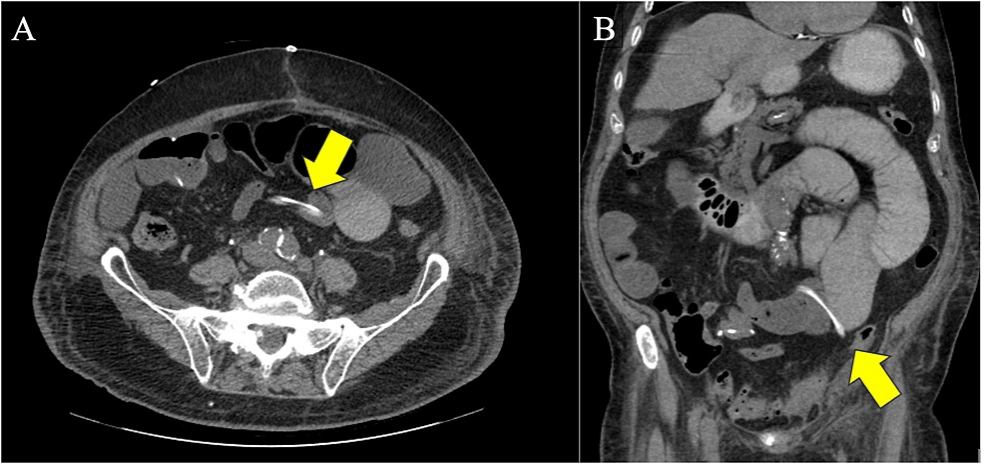
CTAP without contrast on post-op day 7, indicating a transition point at the LLQ with mild compression from the percutaneous surgical drain (A: Axial, B: Coronal). CTAP: CT Abdomen and Pelvis; LLQ: Left lower quadrant.

**Figure 2:**
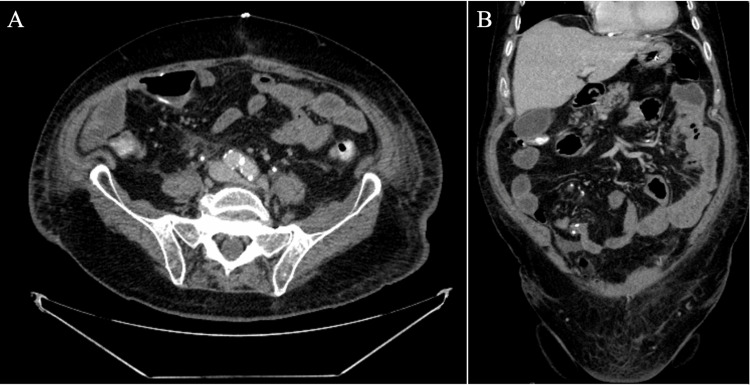
CTAP with IV contrast on post-op day 10 demonstrating resolution of SBO and progression of enteric contrast into the rectum (A: Axial, B: Coronal). CTAP: CT Abdomen and Pelvis; SBO: Small Bowel Obstruction.

The NGT was removed on post-operative day 2 due to a low output of 50 cc in 24 hours, with the patient reporting minor flatus and managing to ambulate around the hospital hallways. By post-operative day 3, the patient could tolerate sips of water but reported hiccups and a lack of appetite. On post-operative day 4, he began to feel more distended, and an examination revealed hypoactive bowel sounds, prompting a repeat abdominal X-ray. Imaging demonstrated multiple segments of dilated air-filled small bowel measuring up to 4.5 cm, concerning for SBO. The decision was made to reinsert the NGT with low continuous wall suction (LCWS) on post-operative day 4 for abdominal decompression, returning 1450 cc of brown bilious fluid. On post-operative day 6, the patient’s abdominal symptoms resolved, and he began passing flatus again, prompting a NGT clamp trial followed by NGT removal. However, that night, the patient experienced increasing abdominal pain and distension, accompanied by nausea and 100cc of bilious vomiting. This led to the reinsertion of the NGT, which immediately yielded 800cc of bilious fluid. A CT abdomen and pelvis (CTAP) with oral contrast through NGT was ordered and performed on post-operative day 7. This showed a 5.4 cm distension of the small bowel with a transition point in the left lower quadrant (LLQ) indicative of an SBO at the surgical drain site. Per radiology, the surgical drain is seen traversing the small bowel loop at the transition point, causing compression (Figure 1). General surgery was consulted, and the drain was removed. A follow-up abdominal X-ray on post-operative day 8 revealed a slight reduction in the caliber of the dilated small bowel, and a confirmatory CT scan on post-operative day 10 demonstrated interval resolution of the SBO (Figure 2). Subsequently, the NGT was removed on post-operative day 11, and the patient could tolerate a clear liquid diet, progressing to a low-residue diet before being discharged on January 31, 2023.

## Discussion

This case highlights a rare but potentially severe complication of surgical drain placement: the development of SBO. Although SBO is a common gastrointestinal complication post major abdominal surgeries, including urological surgeries, SBO caused by surgical drain placement has only been reported a few times. Most of the published case reports are from general surgery cases involving colorectal procedures that involve loops of bowels wrapping around the drain [4]. In urologic surgeries, two cases of pelvic drains related to SBO after robotic-assisted laparoscopic radical prostatectomy have been described [5]. However, the occurrence of SBO following open radical cystectomy due to drain placement has not been previously reported. This case emphasizes the need for a more comprehensive evaluation of the use of prophylactic drain placement after surgery and a higher index of suspicion for SBO in patients with surgical drains who develop post-operative nausea and distention. Early post-operative nausea and distention are most commonly caused by post-operative ileus, which is characterized by hypomobility of the GI tract and usually resolves with conservative management [6]. However, the key is distinguishing post-operative ileus from early SBO, which may require surgical intervention. This patient's physical examination displayed an absence or minimal bowel sounds correlating more with a differential diagnosis of post-operative ileus, but radiological imaging indicated a more SBO-like diagnosis. This situation demonstrates the need for a higher index of suspicion for SBO and further radiologic work-up. Systematic reviews on post-operative ileus have shown that CT scans with oral Gastrografin contrast have high sensitivity and specificity for differentiating post-operative ileus from other conditions [7]. Timely radiological imaging alongside a thorough clinical examination can better assist with the diagnosis seen in this patient's case. 

Another factor that should be reviewed in this case is using prophylactic surgical drains after urological procedures. The use of prophylactic surgical drains has been a common practice for centuries due to their relative safety and low-risk profile. However, there is an ongoing debate among physicians about using prophylactic surgical drains, as no standardized guidelines surround this topic. The primary benefits of a surgical drain include the removal of excess intraperitoneal fluid that can accumulate after surgery and lead to complications like infection, wound dehiscence, and seroma formation. Moreover, a surgical drain can facilitate the earlier detection and management of potential complications, such as bleeding or leakage from surgical anastomoses, which are critical in urological surgeries for the timely detection of urinary leaks. Untreated urinary leakage can cause severe complications, including chemical peritonitis, urinoma formation, and sepsis [8]. Despite being a common practice for many years, the placement of surgical drains carries inherent risks, such as infection, bleeding, fluid and electrolyte imbalances, pain, discomfort, and, in rare cases, mechanical SBO. Randomized trials have been conducted to evaluate the effectiveness of prophylactic drain placement during radical cystectomy, with one study by Özdemir AT et al. (2013) showing no significant difference in hospital stay duration, intestinal peristalsis, renal function, or post-operative complications between patients who received a pelvic drain and those who did not [9]. Similar results were also found in studies on prophylactic drain placement after radical prostatectomy [10-11]. This case report highlights the importance of considering the risks and benefits of prophylactic surgical drain placement on a case-by-case basis, considering factors such as the patient's overall health and the complexity of the surgical procedure [4]. 

## Conclusions

It is crucial to carefully evaluate the advantages and disadvantages of using prophylactic surgical drains. In cases where intraoperative drainage tubes are used, the placement and condition of the drain should be closely monitored. If symptoms of post-operative ileus occur, the possibility of SBO should be considered and promptly investigated, including the differential diagnosis of surgical drain-related SBO. Suppose a confirmed SBO or conservative treatment fails to alleviate the patient's abdominal obstructive symptoms before performing an invasive procedure. In that case, removing the percutaneous drain should be considered and carried out. 
